# External validation and extension of the Early Prediction of Functional Outcome after Stroke (EPOS) prediction model for upper limb outcome 3 months after stroke

**DOI:** 10.1371/journal.pone.0272777

**Published:** 2022-08-08

**Authors:** Janne M. Veerbeek, Johannes Pohl, Andreas R. Luft, Jeremia P. O. Held

**Affiliations:** 1 Department of Neurology, University Hospital Zurich and University of Zurich, Zurich, Switzerland; 2 cereneo, Center for Neurology and Rehabilitation, Vitznau, Switzerland; Seoul National University Bundang Hospital, REPUBLIC OF KOREA

## Abstract

**Objective:**

The ‘Early Prediction of Functional Outcome after Stroke’ (EPOS) model was developed to predict the presence of at least some upper limb capacity (Action Research Am Test [ARAT] ≥10/57) at 6 months based on assessments on days 2, 5 and 9 after stroke. External validation of the model is the next step towards clinical implementation. The objective here is to externally validate the EPOS model for upper limb outcome 3 months poststroke in Switzerland and extend the model using an ARAT cut-off at 32 points.

**Methods:**

Data from two prospective longitudinal cohort studies including first-ever stroke patients admitted to a Swiss stroke center were analyzed. The presence of finger extension and shoulder abduction was measured on days 1 and 8 poststroke in Cohort 1, and on days 3 and 9 in Cohort 2. Upper limb capacity was measured 3 months poststroke. Discrimination (area under the curve; AUC) and calibration obtained with the model were determined.

**Results:**

In Cohort 1 (N = 39, median age 74 years), the AUC on day 1 was 0.78 (95%CI 0.61, 0.95) and 0.96 (95%CI 0.90, 1.00) on day 8, using the model of day 5. In Cohort 2 (N = 85, median age 69 years), the AUC was 0.96 (95%CI 0.93, 0.99) on day 3 and 0.89 (95% CI 0.80, 0.98) on day 9. Applying a 32-point ARAT cut-off resulted in an AUC ranging from 0.82 (95%CI 0.68, 0.95; Cohort 1, day 1) to 0.95 (95%CI 0.87, 1.00; Cohort 1, day 8).

**Conclusions:**

The EPOS model was successfully validated in first-ever stroke patients with mild-to-moderate neurological impairments, who were independent before their stroke. Now, its impact on clinical practice should be investigated in this population. Testing the model’s performance in severe (recurrent) strokes and stratification of patients using the ARAT 32-point cut-off is required to enhance the model’s generalizability and potential clinical impact.

## Introduction

Several studies have shown that upper limb recovery is highly predictable early after stroke when multivariable prediction models are used [1]. One of these models is the Early Prediction of Functional Outcome (EPOS) model developed by Nijland and colleagues in 2010, in which active finger extension and shoulder abduction were assessed within 72 hours and on days 5 and 9 after stroke to predict upper limb outcome at 6 months in a sample of first-ever ischemic stroke patients [2]. Upper limb outcome was assessed using the Action Research Arm Test (ARAT) [3]. The ARAT is a capacity-based measurement instrument that is recommended for stroke rehabilitation [4] and research [5]. The total score of this ordinal scale ranges from 0 (‘no upper limb capacity’) to 57 (‘full upper limb capacity’). In the EPOS prediction model for upper limb outcome, the ARAT was dichotomized into <10/57 (defined as ‘no dexterity’, unfavorable outcome) and ≥10/57 (defined as ‘some dexterity’, favorable outcome) [2]. Active finger extension was assessed by the finger extension item of the upper extremity subscale of the Fugl-Meyer Assessment (FMA-UE FE), and active shoulder abduction was assessed by the shoulder abduction item of the Motricity Index upper extremity subscale (MI-UE SA). Patients who had at least some active finger extension (FMA-UE FE score of ≥1/2) and at least a visible or palpable contraction of the shoulder abduction muscles (MI-UE SA score of ≥9/33) within 72 hours after stroke onset had a probability of 98% to regain some dexterity at 6 months. Patients who did not fulfill these criteria had a probability of 25%. If finger extension and shoulder abduction were also absent on day 5 and/ or 9 poststroke this probability decreased to 14% [2].

Although the development study showed a good performance of the EPOS model and the tests are easy to obtain in clinical practice, the model is not ready for implementation in clinical practice yet. The next step in prognosis research is testing its performance in an independent sample [6, 7]. This so-called ‘external validation’ is needed to evaluate whether the model’s performance remains in cohorts with a different case-mix, that are recruited in another country (with a different health-care system) or in another setting, and at a different time point [7]. A commonly observed phenomenon in external validation studies is that the performance in the new cohort is less satisfying than the performance in the cohort in which the model was developed [8–10], which indicates overfitting. In the case of insufficient performance, the model’s clinical relevance is low and the model in its current form cannot move to the next stage in prognosis research, in which its clinical impact is tested [7, 11, 12].

In the EPOS study, a 6-month outcome time point was selected. However, patients’ behavior mainly changes within the first few months after stroke [13, 14] and most stroke trials use a 3-month endpoint. We therefore chose to use the 3-month endpoint, which is the end of the early subacute phase [5, 13]). This time point matches the endpoint of the Predicting potential for upper limb recovery 2 (PREP2) model [15].

The PREP2 model was a further development, but not a formal external validation, of the Predicting potential for upper limb recovery (PREP) model [16]. In PREP2, the patient’s ability to perform active shoulder abduction and finger extension at day 3 poststroke was the starting point of the classification and regression tree for predicting upper limb outcome as assessed by the ARAT. In this model. shoulder abduction and finger extension (‘SAFE’) were not measured using the FMA-UE FE and MI-UE SA as done in the EPOS model, but with the Medical Research Council scale (score range 0–5 for each movement) and the sum of these scores was taken. A SAFE score of at least 5/10 was defined as positive, and less than 5 points as negative. Apart from SAFE, other model variables were the patient’s age, National Institutes of Health Stroke Scale (NIHSS) score, and the presence of motor evoked potentials in response to transcranial magnetic stimulation (TMS). The PREP2 model not only contains more parameters as compared to the EPOS model, but its outcome is also more nuanced by using four ARAT strata: ‘excellent’ (50–57 points), ‘good’ (34–48 points), limited (13–31 points), and ‘poor’ (0–9 points) upper limb outcome. EPOS has been criticized for using a dichotomized outcome [1]. The favorable outcome range of EPOS (ARAT 10 to 57 points) is too wide and could hamper guiding clinical decision making for patients who have a predicted ARAT-outcome of more than 10 points. Jordon et al. recently reported that 84% of the patients poststroke had an ARAT score of at least 10 points at 3 months [17].

Therefore, the primary objective here was to carry out a geographical and temporal external validation of the EPOS model for the upper limb using a Swiss cohort using slightly different predictor time points (days 1, 3, 8, and 9 poststroke) and a 3-month endpoint. We hypothesized that the performance of the EPOS model on days 1, 3, 8, and 9 poststroke would be lower than in the development cohort due to a different case-mix, the widespread use of thrombectomy after the positive clinical trials in 2015 [18], and a different time schedule of predictor and outcome assessment. However, we expected the application of the model on days 3, 8, and 9 poststroke to be acceptable. The secondary aim was to investigate whether the EPOS model can predict upper limb outcome with an ARAT cut-off of 32/57 points, which distinguishes between the ‘excellent’ and ‘good’ vs. the ‘limited’ and ‘poor’ outcome categories of the PREP2 model.

## Materials and methods

### Design

This study included data from two prospective longitudinal cohort studies. Validation Cohort 1 comes from a study specifically designed for the external validation of the EPOS model. Between 15 October 2017 and 14 November 2019, patients consecutively admitted to the Department of Neurology of the University Hospital Zurich (Switzerland) with a stroke were screened. This hospital has a comprehensive stroke center treating 1100 acute stroke patients annually. Patients from Cohort 1 had three study visits. The first took place within 48 hours after symptom onset, the second on day 7±2 and the third on day 90±10 poststroke. The last visit of the final enrolled patient was performed in January 2020. Cohort 2 was collected as part of a study aiming to profile the natural course of physical activity and upper limb use poststroke. Recruitment took place between 1 September 2018 and 31 December 2020 according to the same screening procedures as for Cohort 1. This study included six study visits, namely on days 3±2, 10±2, 28±4, 90±7, and 365±14 poststroke, as well as at rehabilitation discharge. For thiswork, only data collected on days 3±2, 10±2, and 90±7 were used.

Ethical approval from the cantonal ethics committee Zurich was obtained before study start (BASEC identifiers 2017–00889 and 2017–01070) and the studies were prospectively registered (ClinicalTrials.gov Identifiers NCT03287739 and NCT03522519). Secondary data analysis for Cohort 2 was approved by the aforementioned ethics committee (BASEC identifier 2020–00218). Reporting adhered to the STROBE [19] and TRIPIOD statements [20].

### Participants

The inclusion and exclusion criteria of the two validation cohorts are presented in Table 1. To facilitate a comparison between these two validation cohorts and the development cohort, the key characteristics of the development study by Nijland and colleagues [2] are also shown.

**Table 1:** Key characteristics of the development and validation studies.

Characteristic	Development cohort [2]	Validation cohort 1	Validation cohort 2
Recruitment period	02/2007–01/2009	10/2017–11/2019	09/2018–12/2020
Setting	9 acute hospital stroke units in the Netherlands	1 acute hospital stroke center in Switzerland	1 acute hospital stroke center in Switzerland
Inclusion criteria	(1) first-ever ischemic anterior circulation stroke (2) ≥18 years (3) mono- or hemiparesis <72 hours (4) premorbid Barthel Index ≥19 (5) no severe deficits in communication, memory, or understanding that impede proper measurement performance (6) signed informed consent	(1) first-ever unilateral ischemic stroke <48 hours, confirmed by MRI-DWI and/ or CT (2) ≥18 years (3) National Institutes of Health Stroke Scale arm ≥1 (4) prestroke modified Rankin Scale ≤2 (5) able to follow one-staged commands (6) informed consent after participants’ information	(1) first-ever ischemic or hemorrhagic stroke, confirmed by MRI-DWI and/ or CT (recurrent strokes are allowed when already included in this study after a first-ever stroke) (2) ≥18 years (3) Motricity Index Upper Extremity subscale <100 (4) prestroke modified Rankin Scale ≤2 (5) written informed consent of the patient or its legal representative after participants’ information
Exclusion criteria	Not formulated	(1) neurological or other diseases affecting the upper limb(s) before stroke (2) intravenous line in the upper limb(s) that limited assessment (3) contra-indications on ethical grounds (vulnerable persons) (4) expected or known non-compliance, severe drug and/ or alcohol abuse	(1) neurological or other diseases affecting upper limb use and/ or physical activity before stroke (2) contra-indications on ethical grounds (vulnerable persons) (3) known or suspected non-compliance, drug and/ or alcohol abuse
Outcome(s)	ARAT: <10 vs. ≥10, 6 months poststroke	ARAT: <10 vs. ≥10, 3 months poststroke ARAT: <32 vs. ≥32, 3 months poststroke	ARAT: <10 vs. ≥10, 3 months poststroke ARAT: <32 vs. ≥32, 3 months poststroke
Predictors*	FE (item from FMA-UE): <1 vs. ≥1 SA (item from MI-UE): <9 vs. ≥9	FE (item from FMA-UE): <1 vs. ≥1 SA (item from MI-UE): <9 vs. ≥9	FE (item from FMA-UE): <1 vs. ≥1 SA (item from MI-UE): <9 vs. ≥9

Data from the development cohort was extracted from the publication by Nijland et al. [2].

*, dichotomized predictors are coded 0 and 1; ARAT, Action Research Arm Test; CT, Computed Tomography; FE, Finger Extension; FMA-UE, Fugl-Meyer Assessment Upper Extremity Subscale; MI-UE, Motricity Index Upper Extremity Subscale; MRI-DWI, Magnetic Resonance Diffusion-Weighted Imaging; SA, Shoulder Abduction.

All patients in the validation cohorts had given written consent for the further use of encrypted health-related data. Patients received medical and rehabilitative treatment according to Swiss national guidelines [21], and local hospital and rehabilitation center protocols. Physical and occupational therapy were problem- and task-oriented and had a repetitive nature.

### Data collection

Experienced, unblinded physical therapy researchers performed the assessments. For both studies, visits 1 and 2 took place during hospitalization, or at the individual location of stay when the patient was discharged before visit 2. The assessment on day 90 took place during an outpatient visit or at the patient’s home.

### Outcome

The dependent variable upper limb capacity 90 days after stroke was measured using the ARAT (score range 0–57) [3, 22] and dichotomized into <10 points (unfavorable outcome, no upper limb capacity) and ≥10 points (favorable outcome, some upper limb capacity) to externally validate the EPOS model [2]. For the secondary aim of this study, the ARAT was dichotomized into <32 points (‘poor’ or ‘limited’ outcome, according to PREP2 [15]) and ≥32 points (‘good’ or ‘excellent’ outcome, according to PREP2 [15]).

### Predictors

The two independent variables in the EPOS model were the assessment of the presence of some finger extension and voluntary activation of the shoulder abductors [2]. To assess finger extension, the finger extension item of the FMA-UE was used (score range 0–2, higher scores being better) [23], in which mass finger extension is tested and dichotomized into <1 and ≥1 [2]. A score of 0 means that there is no voluntary movement and 1 means that the movement can partially be performed. Shoulder abduction was measured using the shoulder abduction item of the MI-UE (score range 0–33, higher scores being better) [24] and dichotomized into <9 and ≥9 [2]. A score of 0 means ‘no movement’ and a score of 9 ‘palpable contraction in the muscle, but no movement’ [24].

Data obtained to characterize the current patient samples included patient demographics, stroke event data, NIHSS [25, 26], FMA-UE [23], MI [24], sitting balance item of the Trunk Control Test [24], Functional Ambulation Categories [27–29], and modified Rankin Scale [30].

### Sample size

A formal sample size calculation was not performed, because approaches to determine the minimum number of patients for validating a multivariable logistic regression model are lacking [31]. Therefore, all available patients were included in this study.

### Statistical analysis methods

Data were entered in an electronic case report form (Cohort 1: secuTrial, interActive Systems, Berlin, Germany; Cohort 2: REDCap, Vanderbilt University Medical Center, US) and 100% cross-validated. Patients who died before the day 90 visit were excluded from all analyses.

Baseline characteristics were analyzed by nonparametric descriptive statistics (median [quartile 1 –quartile 3] and frequencies). The EPOS model was externally validated for days 2, 5, and 9 with data of the validation cohorts collected at study visits 1 and 2. Data from Cohort 1 and 2 were not pooled, because the assessment time points of the independent variables differed (Mann-Whitney U, p<0.001). Visit 1 data from Cohorts 1 and 2 were used to validate the model for day 2. The visit 2 data of Cohort 1 were used for both the day 5 and day 9 model, and the visit 2 data of Cohort 2 for the model for day 9. Differences between both cohorts and between patients with and without missing data points for predictors and/ or outcomes were tested with nonparametric statistics, namely the Mann-Whitney U for ordinal-scaled variables and the Chi-squared test for nominal-scaled variables.

The following beta values were extracted for external validation of the EPOS model for upper limb outcome [2]:

$$Day\;2:\;P=1/1+({exp}^{\left[-(-1.119+2.807*FMA-UE\;FE+2.149*MI-UE\;SA)\right]})(presence=1,\;absence=0)$$ (1)

$$Day\;5:\;P=1/1+({exp}^{\left[-(-1.874+3.070*FMA-UE\;FE+3.075*MI-UE\;SA)\right]})(presence=1,\;absence=0)$$ (2)

$$Day\;9:\;P=1/1+({exp}^{\left[-(-1.815+3.224*FMA-UE\;FE+2.449*MI-UE\;SA)\right]})(presence=1,\;absence=0)$$ (3)

For the main analysis, imputed data sets were used. Multiple imputation with 100 imputations and 5 iterations was applied to estimate missing data on predictors using the predictive mean matching algorithm. Data used for predictor imputation included the raw scores of the following variables’: shoulder abduction at visits 1 and 2, finger extension at visits 1 and 2, NIHSS at visit 1, lower extremity subscale of the MI at visit 1, sitting balance item of the Trunk Control Test at visit 1, dominant side affected (yes/ no), affected side (left/ right), Bamford classification, gender (male/ female), and ARAT at visit 3 of all included subjects. Thereafter, subjects with a missing outcome assessment were dropped (i.e. “multiple imputation, then deletion”) [32, 33]. Imputation was performed with the R package ‘Multivariate Imputation by Chained Equations (mice)’ [34].

Discrimination was analyzed by the area under the receiver operating characteristic curve (AUC). By using the AUC, the ability of the EPOS model to distinguish between patients who regained some upper limb capacity at 3 months poststroke and those who did not was determined [31]. An AUC of 0.5 indicates that the model cannot discriminate and a value of 1.00 means perfect discrimination. In this work, an AUC of >0.75 was defined as clinically useful [35] and the AUC’s 95% confidence interval [CI] was calculated using DeLong’s method. Calibration-in-the-large was assessed by calibration plots that displayed the agreement between the predicted probabilities by the EPOS model on the x-axis and the observed probabilities in our sample on the y-axis [31]. Perfect calibration is indicated by a 45^o^ line. The closer the calibration points are to this line, the better the calibration. The classification measures sensitivity, specificity, and positive and negative predictive values with their corresponding 95%CI (i.e., exact binomial confidence limits) were calculated to assess clinical utility. Furthermore, the ‘no information rate’ was determined; this value reflects the size of the most common outcome class in the sample (i.e., the outcome category on the dichotomized ARAT with the highest prevalence). In a sensitivity analysis, the abovementioned procedures were repeated with the two non-imputed data sets.

For the secondary aim of this study, the afore-mentioned analyses were repeated using an ARAT cut-off at 32-points.

RStudio software with R version 3.6.3 was used for the statistical analyses [36] and the level of statistical significance was set to <0.05.

### Data disclosure statement

The dataset is included as a supporting information file (S1 Appendix).

## Results

**Fig 1:**
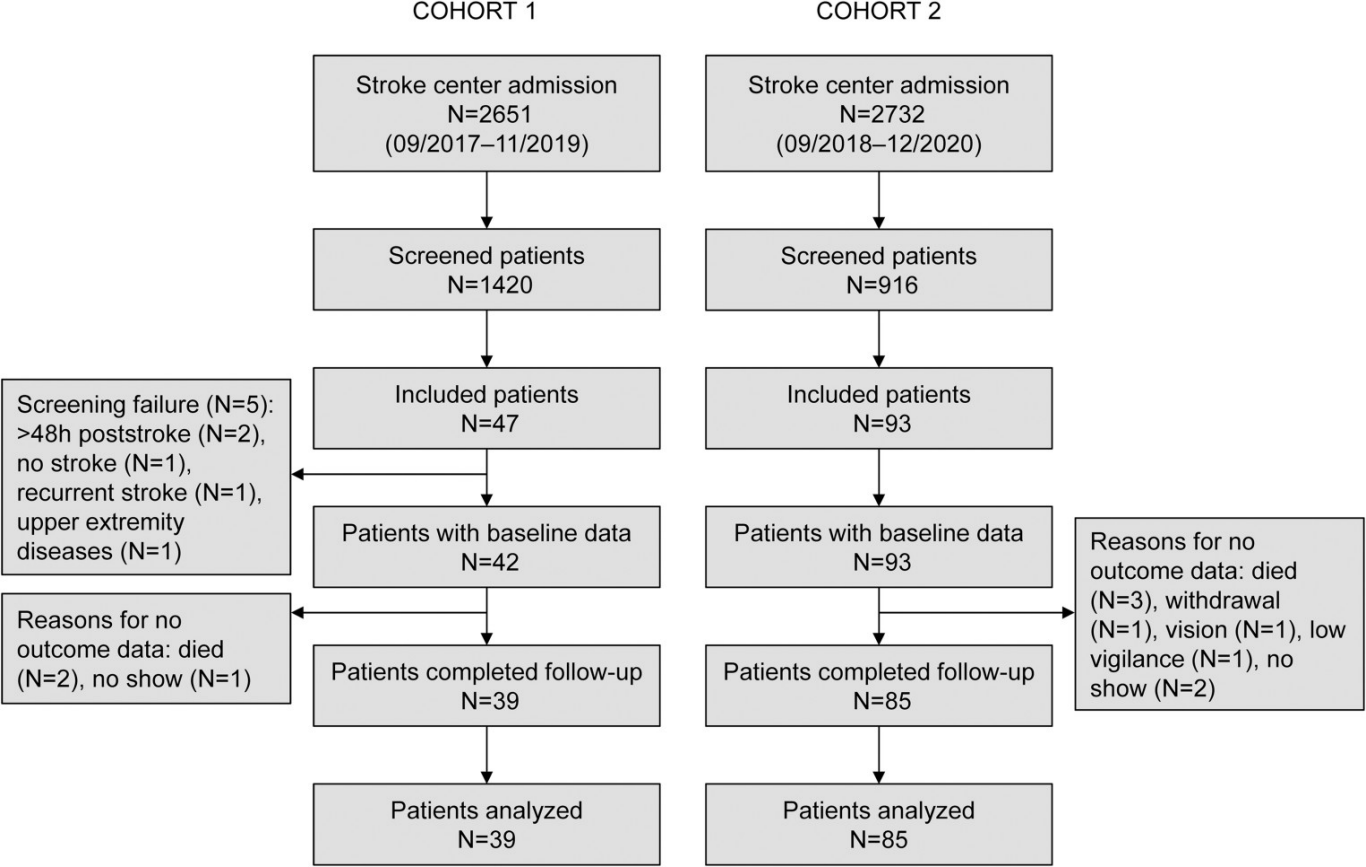
Flow chart for the validation cohorts.

Fig 1 displays the patient flow for both cohorts. The patient characteristics of the validation and the development cohorts can be found in Table 2 and S1 Table. The median ARAT score amounted to 38 (5–48) and 38 (10–57) points on day 90 in Cohort 1 and 2, respectively. No predictor data were missing for visit 1. Predictor data of visit 2 were missing in two patients in Cohort 1 (discharge N = 1, transfer to intensive care N = 1) and six in Cohort 2 (discharge N = 5, withdrawal N = 1). Outcome data were missing in one and three patients, respectively. Comparing patients with and without missing data did not reveal significant differences at baseline (S2 Table).

**Table 2:** Characteristics of included patients who were analyzed for model development and external validation.

Characteristic	Development cohort [2]	Validation cohort 1		Validation cohort 2		P-value Cohort 1 vs. Cohort 2
Patients with outcome data	(N = 156)	(N = 39)	Missing data, N (%)	(N = 85)	Missing data, N (%)	
Age, years	66.47 (14.43)*	74 (69–77)†	0 (0)	69 (60–77)†	0 (0)	*0*.*035*
Female‡	87 (55.8)	13 (33.3)	0 (0)	41 (48.2)	0 (0)	0.536
Affected hemisphere, left‡	69 (44.2)	13 (33.3)	0 (0)	44 (51.8)	0 (0)	0.245
Type of stroke‡			0 (0)		0 (0)	*0*.*004*
Ischemic	156 (100)	39 (100)		66 (77.6)		
Hemorrhagic	0 (0)	0 (0)		19 (22.4)		
Bamford classification‡			0 (0)		0 (0)	0.727
LACS	79 (50.6)	16 (41)		38 (44.7)		
PACS	50 (32.1)	12 (30.7)		28 (32.9)		
TACS	27 (17.3)	11 (28.2)		19 (22.4)		
Thrombolysis, yes‡	39 (25)	15 (38.5)	0 (0)	17 (20)	0 (0)	*0*.*020*
Thrombectomy, yes‡	N/A	16 (41)	0 (0)	27 (31.7)	0 (0)	0.359
Time poststroke						
Model day 2 (days)	2.26 (1.28)*	1.07 (0.74–1.37)†	0 (0)	3 (2–4)†	0 (0)	*<0*.*001*
Model day 5 (days)	5.48 (1.40)*	7.85 (7.38–8.31)†	2 (5.1)	N/A	N/A	N/A
Model day 9 (days)	9.02 (1.81)*	7.85 (7.38–8.31)†	2 (5.1)	9 (8–10)†	2 (2.4)	*<0*.*001*
Clinical scales baseline						
NIHSS (0–42)†	7 (4–14)	9 (5.5–13.5)	0 (0)	7.5 (5–11.25)	1 (1.2)	*0*.*036*
Cognitive disturbance, yes‡						
Inattention	63 (40.4)	18 (46.2)	0 (0)	23 (27.1)	1 (1.2)	0.050
Disorientation	37 (23.7)	14 (35.9)	0 (0)	22 (25.9)	0 (0)	0.241
Sensation deficits, yes‡	N/A	21 (53.8)	0 (0)	40 (47.1)	0 (0)	0.672
Visual impairment, yes‡						
Hemianopia	42 (26.9)	6 (15.4)	0 (0)	24 (28.2)	0 (0)	0.140
Deviation conjugee	34 (21.8)	13 (33.3)	0 (0)	16 (18.8)	0 (0)	0.140
MI-UE (0–100)†	39 (0–76)	37 (4.5–61)	0 (0)	50 (18–65)	0 (0)	0.087
MI-LE (0–100)†	53 (23–83)	37 (20.5–60.5)	1 (2.6)	42 (28–75)	0 (0)	0.061
FMA-UE (0–66)†	21 (4–56)	10.5 (4–23.5)	1 (2.6)	22 (7–37)	0 (0)	*0*.*006*
FAC (0–5)†	1 (0–3)	0 (0–0)	0 (0)	0 (0–2)	0 (0)	*<0*.*001*
ARAT (0–57)†	1.5 (0–41)	N/A	N/A	N/A	N/A	N/A
mRS (0–5)†	N/A	5 (4–5)	0 (0)	4 (4–5)	0 (0)	*0*.*004*
BI (0–20)†	8 (3–14)	N/A	N/A	N/A	N/A	N/A
Predictors						
Model day 2						
FE, yes‡	82 (52.6)	21 (53.8)	0 (0)	54 (63.5)	0 (0)	0.224
SA, yes‡	104 (66.7)	28 (71.8)	0 (0)	69 (81.2)	0 (0)	0.182
Model day 5						
FE, yes‡	N/R	22 (56.4)	2 (5.1)	N/A	N/A	N/A
SA, yes‡	N/R	29 (74.4)	2 (5.1)	N/A	N/A	N/A
Model day 9						
FE, yes‡	N/R	22 (56.4)	2 (5.1)	57 (67.1)	5 (5.9)	0.205
SA, yes‡	N/R	29 (74.4)	2 (5.1)	72 (84.7)	5 (5.9)	0.145
Outcome			0 (0)		0 (0)	
ARAT (0–57)†	N/R	38 (5–48)		38 (10–57)		0.093
Subgroup <10	N/R	2 (0–4.25)		0 (0–0)		0.069
Subgroup ≥10	N/R	42 (38–52.5)		52 (38–57)		*0*.*046*
Subgroup <32	N/R	2 (0–4.25)		0 (0–10)		0.646
Subgroup ≥32	N/R	42 (38–52.5)		54.5 (41–57)		*0*.*006*
ARAT ≥10‡	110 (70.5)	27 (69.2)		65 (76.5)		0.526
ARAT ≥32‡	N/R	24 (61.5)		56 (65.9)		0.789
ARAT categorized according to PREP2						*0*.*048*
Poor‡	N/R	12 (30.8)		20 (23.5)		
Limited‡	N/R	3 (7.7)		9 (10.6)		
Good‡	N/R	15 (38.5)		17 (20)		
Excellent ‡	N/R	9 (23.1)		39 (45.9)		

Data from the development cohort was extracted from the publication by Nijland et al. [2].

*, mean (standard deviation)

†, median (quartile 1 –quartile 3)

‡, N (%); ARAT, Action Research Arm Test; BI, Barthel Index; FAC, Functional Ambulation Categories; FE, Finger Extension; FMA-UE, Fugl-Meyer Assessment Upper Extremity Subscale; LACS, Lacunar Stroke; MI-LE, Motricity Index Lower Extremity Subscale; MI-UE, Motricity Index Upper Extremity; mRS, modified Rankin Scale; N, Number; N/A, Not Applicable; N/R, Not Reported; NIHSS, National Institutes of Health Stroke Scale; PACS, Partial Anterior Circulation Stroke; PREP2, Predicting potential for upper limb recovery 2; SA, Shoulder Abduction; TACS, Total Anterior Circulation Stroke.

**Fig 2:**
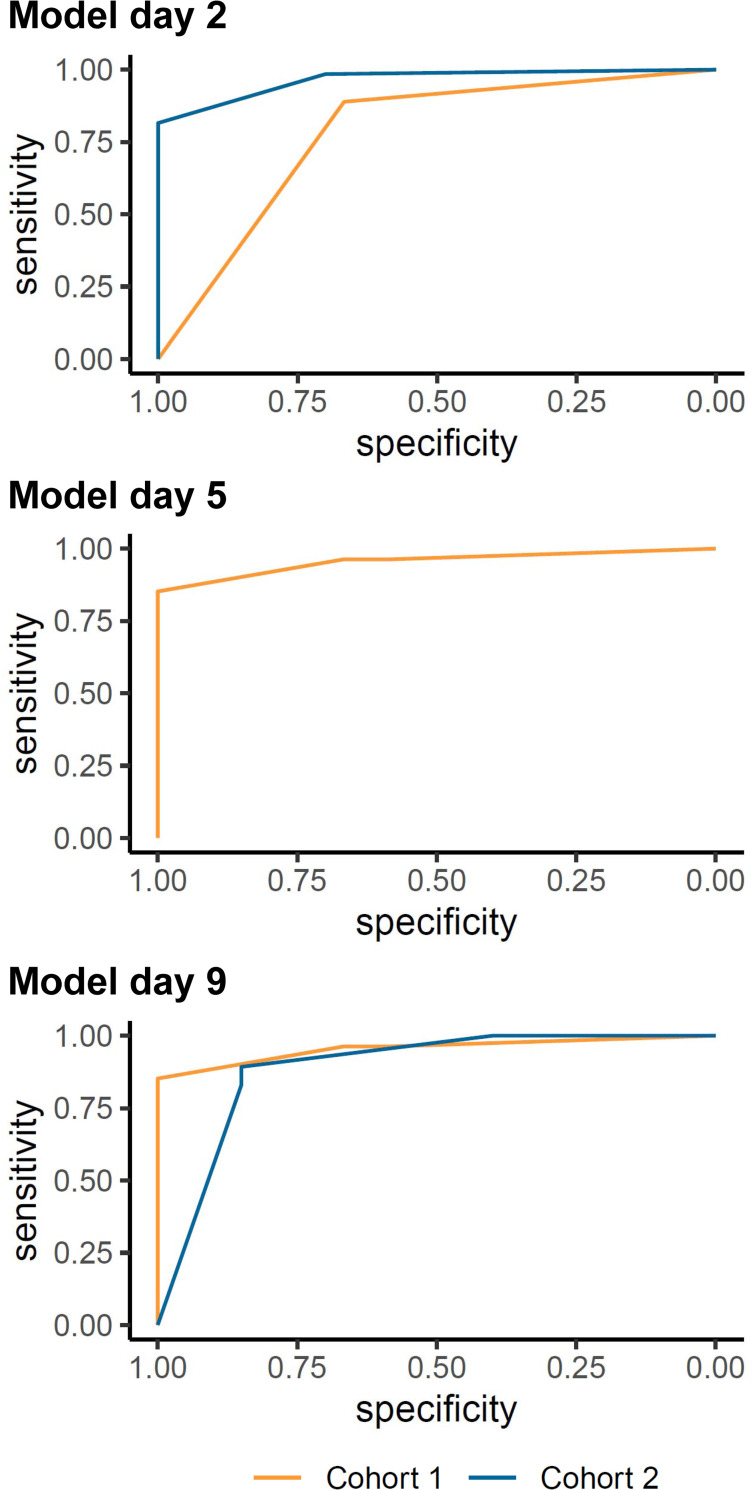
Receiver operator characteristic curves for the external validation of the EPOS model for an ARAT cut-off at 10 points. Analysis based on the imputed data.

Discrimination was acceptable in Cohort 1 with an AUC of 0.778 (95% confidence interval [CI] 0.610, 0.945) on day and excellent in Cohort 2 on day 3 (AUC 0.965 [95% CI 0.935, 0.995]) (Table 3; Fig 2). On day 8, the AUC improved in Cohort 1 to 0.955 (95% CI 0.898, 1.000) and 0.946 (95% CI 0.883, 1.000), using the beta-values from the EPOS model on day 5 and 9, respectively. On day 9, the AUC was 0.889 (95% CI 0.795, 0.981) in Cohort 2.

**Table 3:** Discrimination of the EPOS model in the development and validation cohorts for an ARAT cut-off at 10 and 32 points.

	Development cohort [2]	Validation cohort 1		Validation cohort 2	
ARAT cut-off	10/57	10/57	32/57	10/57	32/57
Model day 2	N = 156	N = 39	N = 39	N = 85	N = 85
Accuracy (95% CI)	N/R	0.82 (0.66, 0.92)	0.79 (0.64, 0.91)	0.92 (0.84, 0.97)	0.84 (0.74, 0.91)
Sensitivity	0.89	0.89 (0.71, 0.98)	0.92 (0.73, 0.99)	0.98 (0.92, 1.00)	1.00 (0.94, 1.00)
Specificity	0.93	0.67 (0.35, 0.90)	0.60 (0.32, 0.84)	0.70 (0.46, 0.88)	0.52 (0.33, 0.71)
Positive predictive value	0.93	0.86 (0.67, 0.96)	0.79 (0.59, 0.92)	0.91 (0.82, 0.97)	0.80 (0.69, 0.89)
Negative predictive value	0.76	0.73 (0.39, 0.94)	0.82 (0.48, 0.98)	0.93 (0.68, 1.00)	1.00 (0.78, 1.00)
No information rate	N/R	0.69 (0.52, 0.83)	0.62 (0.45, 0.77)	0.76 (0.66, 0.85)	0.66 (0.55, 0.76)
P-Value [Acc > NIR]	N/R	0.054	0.014	<0.001	<0.001
AUC (95% CI)	N/R	0.78 (0.61, 0.95)	0.82 (0.68, 0.95)	0.96 (0.93, 0.99)	0.90 (0.83, 0.97)
Model day 5	N = 156*	N = 39	N = 39		
Accuracy (95% CI)	N/R	0.85 (0.69, 0.94)	0.82 (0.66, 0.92)		
Sensitivity	0.95	0.96 (0.81, 1.00)	1.00 (0.86, 1.00)		
Specificity	0.83	0.58 (0.28, 0.85)	0.53 (0.27, 0.78)		
Positive predictive value	0.93	0.84 (0.66, 0.95)	0.77 (0.59, 0.90)		
Negative predictive value	0.86	0.88 (0.47, 1.00)	1.00 (0.63, 1.00)		
No information rate	N/R	0.69 (0.52, 0.83)	0.62 (0.45, 0.77)		
P-Value [Acc > NIR]	N/R	0.023	0.005		
AUC (95% CI)	N/R	0.96 (0.90, 1.00)	0.95 (0.87, 1.00)		
Model day 9	N = 156*	N = 39	N = 39	N = 85	N = 85
Accuracy (95% CI)	N/R	0.85 (0.69, 0.94)	0.82 (0.66, 0.92)	0.86 (0.77, 0.92)	0.75 (0.65, 0.84)
Sensitivity	0.95	0.96 (0.81, 1.00)	1.00 (0.86, 1.00)	1.00 (0.94, 1.00)	1.00 (0.94, 1.00)
Specificity	0.83	0.58 (0.28, 0.85)	0.53 (0.27, 0.79)	0.40 (0.19, 0.64)	0.28 (0.13, 0.47)
Positive predictive value	0.93	0.84 (0.66, 0.95)	0.77 (0.59, 0.90)	0.84 (0.74, 0.92)	0.73 (0.61, 0.82)
Negative predictive value	0.86	0.88 (0.47, 1.00)	1.00 (0.63, 1.00)	1.00 (0.63, 1.00)	1.00 (0.63, 1.00)
No information rate	N/R	0.69 (0.52, 0.83)	0.62 (0.45, 0.77)	0.76 (0.66, 0.85)	0.66 (0.55, 0.76)
P-Value [Acc > NIR]	N/R	0.023	0.005	0.023	0.041
AUC (95% CI)	N/R	0.96 (0.90, 1.00)	0.95 (0.87, 1.00)	0.89 (0.80, 0.98)	0.86 (0.77, 0.95)

Data from the development cohort was extracted from the publication by Nijland et al. [2].

*, Not explicitly stated; Acc, Accuracy; ARAT, Action Research Arm Test; AUC, Area Under the Curve; CI, Confidence Interval; N/R, Not Reported; NIR, No Information Rate.

**Fig 3:**
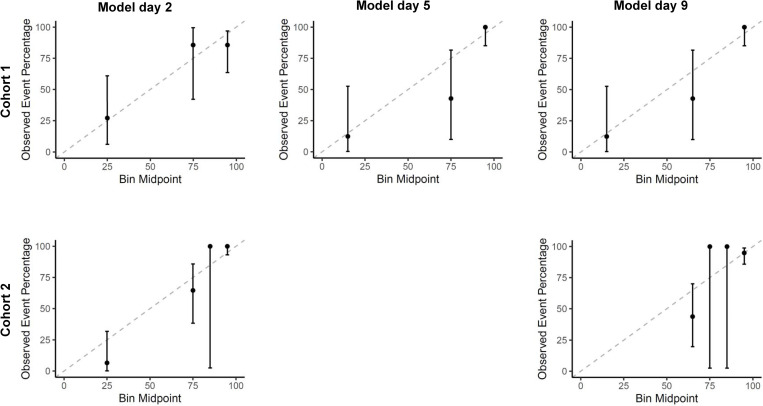
Calibration plots for the external validation of the EPOS model for an ARAT cut-off at 10 points. Analysis based on the imputed data. The model on day 5 was not externally validated in Cohort 2. The dotted line indicates perfect calibration, meaning that the predicted probabilities by the EPOS model (x-axis) and the observed probabilities in our sample (y-axis) are similar. ARAT, Action Research Arm Test.

For all time points, the sensitivity outperformed the model’s specificity and the positive and negative predictive values were high. The sensitivity ranged from 0.89 (95%CI 0.71, 0.98; Cohort 1, day 1) to 1.00 (95%CI 0.94, 1.00; Cohort 2, day 9), and the specificity from 0.40 (95%CI 0.19, 0.64; Cohort 2, day 9) to 0.70 (95%CI 0.46, 0.88; Cohort 2, day 3). The positive predictive value ranged from 0.84 (95%CI 0.66, 0.95; Cohort 1, day 8) to 0.91 (95%CI 0.82, 0.97; Cohort 2, day 3) and the negative predictive value from 0.73 (95%CI 0.39, 0.94; Cohort 1, day 1) to 1.00 (95%CI 0.63, 1.00; Cohort 2, day 9). Full information on the classification measures is reported in Table 3, and the calibration plots are presented in Fig 3. An overview of the predicted and actual outcome categories is presented in S4 Table. The sensitivity analysis of the raw data did not lead to different results (S3 Table, S1 and S2 Figs).

**Fig 4:**
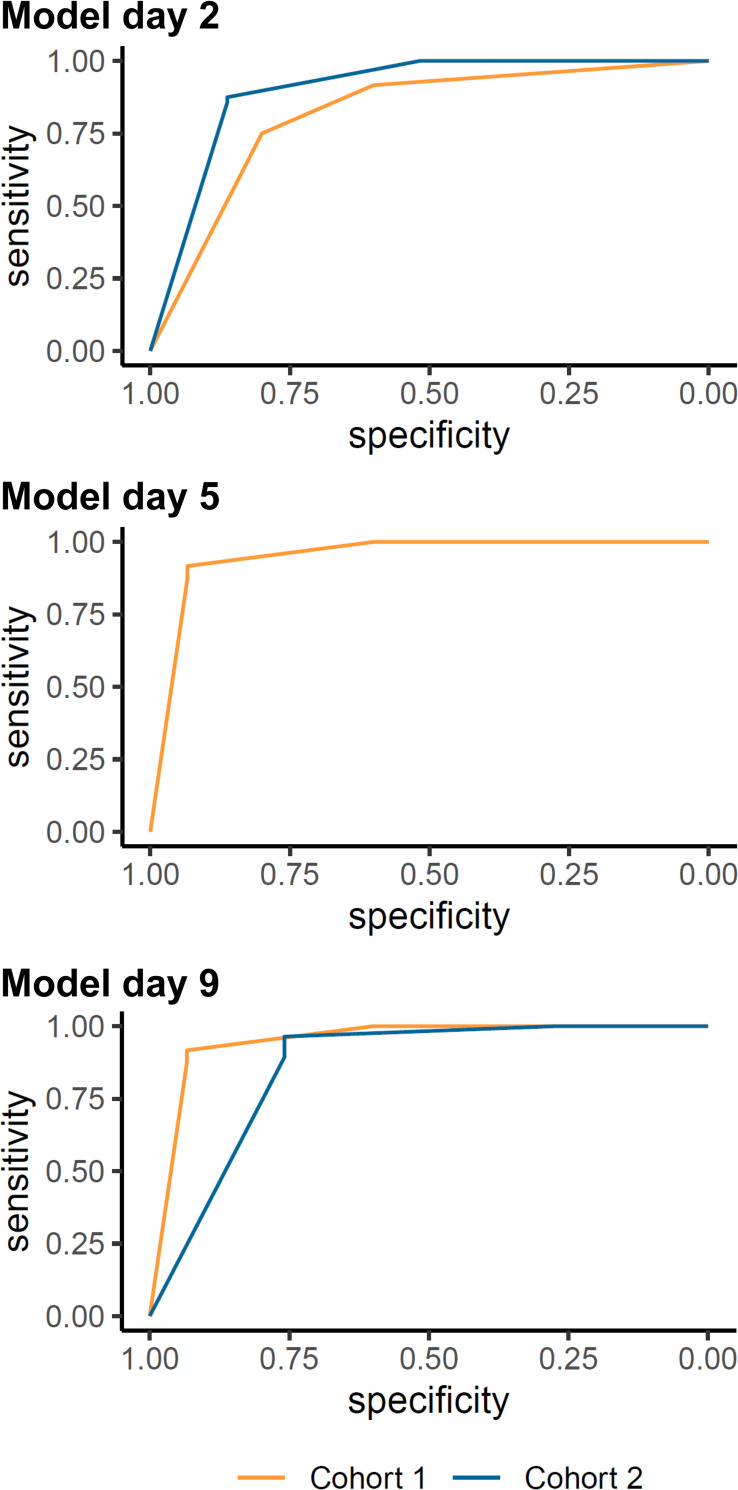
Receiver operator characteristic curves for the external validation of the EPOS model for an ARAT cut-off at 32 points. Analysis based on the imputed data. The model for day 5 was not externally validated in Cohort 2. ARAT, Action Research Arm Test.

**Fig 5:**
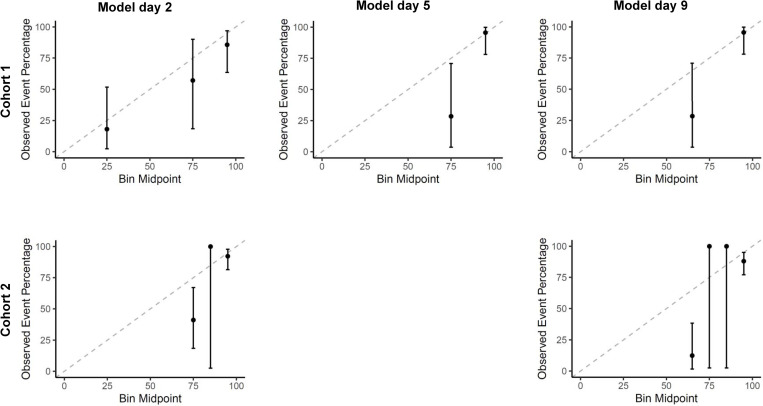
Calibration plots for the external validation of the EPOS model for an ARAT cut-off at 32 points. Analysis based on the imputed data. The model for day 5 was not externally validated in Cohort 2. The dotted line indicates perfect calibration, meaning that the predicted probabilities by the EPOS model (x-axis) and the observed probabilities in our sample (y-axis) are similar. ARAT, Action Research Arm Test.

Testing the EPOS model for upper limb outcome using an ARAT cut-off of 32 points resulted in an AUC in Cohort 1 of 0.82 (95%CI 0.68, 0.95) on day 1, and 0.95 (95%CI 0.87, 1.00) for day 8 using the day 5 and 9 models (Table 3; Fig 4). In Cohort 2, the AUC on day 3 was 0.90 (95%CI 0.83, 0.97) and 0.86 (95%CI 0.77, 0.95) on day 9 (Table 3; Fig 4). Full data on the classification measures are presented in Table 3. The calibration plots are displayed in Fig 5, an overview of the predicted and actual outcome categories is provided in S5 Table, and the sensitivity analysis on the raw data can be found in S6 Table, and S3 and S4 Figs.

## Discussion

To our knowledge, this was the first external validation of the EPOS model for predicting outcome of upper limb capacity after stroke using a dataset that differs in geographical origin and time schedule from the dataset of the development study [2]. Testing the EPOS model in two independent Swiss cohorts showed that it discriminates well between patients with and without at least some upper limb capacity 3 months after stroke, especially when applied between days 3 and 9 after symptom onset. Furthermore, the point estimates of the calibration plots were generally close to the ideal 45^o^ agreement line, except for the patients who had either finger extension or shoulder abduction. In those patients, the EPOS model tended to underestimate the outcome in Cohort 1 on day 1, but overestimated in the same cohort on day 8 and in Cohort 2 for both time points. However, the under- and overestimation were not significant. The clinical utility of the model was good in terms of sensitivity, but moderate in specificity. The positive and negative predictive values were high, which is essential for making individual patient predictions in clinical practice [37]. The ‘favorable outcome’ category (ARAT score of 10 to 57 points) was too broadly defined in the original study. We therefore evaluated a cut-off point of 32 points, similar to the PREP2 study, reflecting a ‘poor’ or ‘limited’ outcome versus a ‘good’ or ‘excellent’ outcome [15]. Results were similar to the 10-point cut-off. Also, the classification measures were comparable, with a good sensitivity, and positive and negative predictive values, but a low-to-moderate specificity. The negative predictive values were even higher using a 32-point ARAT cut-off, indicating that virtually all patients without voluntary finger extension and shoulder abduction early after stroke had an ARAT of <32 points 3 months after stroke onset. The calibration plots showed that the EPOS model tended to overestimate the predicted outcome, which is expected at an ARAT cut-off at 32. As the EPOS model was developed for predicting upper limb outcome using a 10-point cut-off, the resulting probabilities do not fit the 32-point cut-off.

The variations found in the discrimination of the EPOS model with an ARAT cut-off at 10 points in the development and validation cohorts could be attributable to the fact that prediction models generally perform worse in independent cohorts than in the development cohort [7]. Lower performance could also be due to differences in the timing of the predictor assessment: while it was day 2 in the development cohort, our first assessment in Cohort 1 was on day 1 poststroke and on day 3 in Cohort 2. The early time point in Cohort 1 could be a reason for the slightly lower specificity and negative predictive value, acknowledging that neurological deficits are highly dynamic in the (hyper)acute phase. Furthermore, upper limb capacity outcome was measured 3 months poststroke in the validation cohorts, instead of at 6 months in the development study. Although most recovery occurs within the first 3 months poststroke [38, 39], this does not necessarily mean that patients cannot improve further between 3 and 6 months. However, the proportion of patients who had some upper limb capacity was the same or even higher in the validation cohorts at 3 months than in the development cohort at 6 months. It is therefore unlikely that the performance of the model would have been worse if patients in the validation cohorts were measured 6 months after stroke. The observed differences could also be the result of how missing data was handled. Nijland et al. excluded patients with missing FMA-UE data at baseline [2], while we imputed missing predictor data. However, with baseline data of three patients missing, the number of missing data points in the development cohort was small, and it is unlikely that this would have considerably influenced the results. As a comparison, the performance of the EPOS model in our sensitivity analysis with the raw data did not lead to different conclusions. Finally, although patients in the development and validation cohorts had a first-ever stroke resulting in upper limb motor impairments and were independent prior to their stroke, patients in the development cohort were younger and were less likely to have received thrombolysis. Note that the validation cohorts were recruited after the introduction of thrombectomy [18] and 41% (Cohort 1) and ~32% (Cohort 2) of the included patients had received this peracute recanalization therapy. The calibration of the model in the validation cohorts cannot be compared with that in the development cohort, because calibration plots were not reported in the original publication [2].

To advance upper limb prediction models after stroke towards clinical implementation, the EPOS model was deliberately selected for external validation, as it had adequate performance and no special technical equipment was needed to obtain the predictors. This should facilitate the model’s implementation in clinical stroke rehabilitation. There are other upper limb models available that include, for example, assessment of the functional integrity of the corticospinal tract by TMS [15]. However, the need of neurophysiological assessments in addition to clinical assessments for predicting upper limb recovery has been questioned, as they have not shown to improve prediction accuracy beyond the accuracy obtained by simple clinical tests and require large financial investments and time consuming [9, 40]. The proportion of individuals that would have potentially benefitted from TMS (the ‘limited’ category of PREP2) was arguably small in our cohorts (~8% and ~11%, respectively). A quick and low-cost alternative would be reassessing finger extension and shoulder abduction beyond 9 days poststroke. To date, no data on repetitive assessments of these EPOS predictors beyond day 9 poststroke are available. Winters and colleagues monitored the presence of only finger extension in patients who initially did not have voluntary finger extension during the first 6 months poststroke and showed that about 45% of these patients regained finger extension within this time window [41]. These patients regained voluntary finger extension at a median of 4 (Q1: 2, Q3: 8) weeks and had a median ARAT score of 34 (Q1: 19.50, Q3: 45) points at 6 months. These results suggest that serial assessment of finger extension and shoulder abduction throughout the first weeks after stroke could result in improved model performance at a later time point in patients who initially have an unfavorable prognosis.

Our results underline the importance of repeated assessments of upper limb motor function. Our data indicated that for the 10-point cut-off, the false negative rate on day 1 poststroke was ~27% and decreased to 12.5% on day 8 in Cohort 1. In Cohort 2, the false negative rate was ~7% on day 3 and decreased to 0% on day 9. We found the same decline in the false negative rate for the 32-point cut-off. Thus, caution is needed when applying the EPOS model very early after stroke, especially in those patients who initially have no voluntary upper limb motor function (i.e., the ‘unfavorable’ prognosis group). Since prognostic models for upper limb recovery are implemented increasingly [42, 43], this becomes an even more important issue, as the model would misinform therapists and patients in the early false negative prognosis scenario. In the case of an unfavorable prognosis, it recommended to focus on the application of compensational strategies [15]. Consequently, not applying the EPOS model repeatedly within this subgroup can result in an overuse of compensatory approaches that might discourage patients from using their affected upper limb. Another aspect that needs careful attention is that rehabilitation is not withheld from patients with an unfavorable prognosis.

Because the EPOS model using a 10-point ARAT cut-off performed well in our validation cohorts, the next step towards clinical application is testing its impact in the rehabilitation of first-ever stroke patients with mild-to-moderate neurological deficits (median NIHSS 9 [5.5–13.5] in Cohort 1 and 7.5 [5–11.25] in Cohort 2). This step should not be omitted, as although it is assumed that applying a prediction model improves clinical decision making, patients’ outcomes, and cost effectiveness of care [7, 44], its impact remains generally unknown to date. An appropriate impact evaluation would require a comparative design, preferably with a cluster approach on the health care professional or clinic level to avoid contamination. An impact study would also reveal possible adverse consequences, such as reducing or withholding interventions [7]. Considering the high costs and time investments that accompany performing a randomized trial, an alternative is to perform a before and after implementation study [7]. Stinear and colleagues used such a design to investigate the impact of their PREP model [45]. They concluded that after implementation, therapists had changed therapy content and length of stay was decreased. No differences in patient outcomes were found.

The EPOS model with an ARAT cut-off at 32 points is not ready for clinical impact testing. Its discrimination and most of the calibration measures were acceptable, but the calibration plots showed a clear mismatch between predicted and actual probabilities of recovery. This is most likely due to the much higher ARAT cut-off used. The next step in predicting upper limb outcome with a cut-off at 32 points would be to develop a new multivariable logistic regression model, using the FMA-UE FE and MI-UE SA as predictors with new cut-offs in a large, independent sample of stroke patients. We regarded our samples as being too small to develop a new model [46, 47]. Applying the same predictor assessments as in the original EPOS model, the assessments would firstly, allow predict using a 10-point cut-off by using the same predictor cut-offs, and secondly, predict for an outcome cut-off at 32 points using the newly developed model. This extended EPOS model would have the potential to make more specific outcome predictions (e.g., 3 outcome categories using the 10-point and 32-point cut-offs) using simple bedside tests that can be applied globally without the need of special equipment. In this study, the predictors should be repeatedly assessed at fixed time points during the first weeks, to show if and how repeated assessments influence the model’s performance.

Advances in the health care evaluation domain would be using the EPOS model to explain variations seen in outcomes in clinical practice in patients with and without early voluntary shoulder abduction and finger extension [7]. Another topic that needs investigation is the determination of timing and method to communicate the predicted outcome to the patient. Although informing patients and their caregivers regarding the probability for recovery is considered a benefit of a prediction model [2], it is unknown which approach to use in stroke rehabilitation and how it influences the patients’ motivation, especially in the case of an unfavorable prognosis [48, 49].

### Limitations

Both validation cohorts included first-ever stroke patients with mild-to-moderate neurological impairments at baseline, without considerable pre-existing disabilities, and most of the patients had suffered an ischemic stroke. This hampers generalization of the EPOS model to patients with a pre-stroke modified Ranking Scale score of >2, a recurrent or hemorrhagic stroke, and/ or severe neurological impairments. Furthermore, the ARAT was not measured at baseline. Some of the patients may have had a baseline ARAT score of 10 points or more. However, the initial upper limb motor impairment in the validation cohorts as assessed with the FMA was less or equal to the one in the original cohort, in which patients had a median baseline ARAT of 1.5 points. In addition, not using the ARAT as an inclusion criterion fitted the development study. Although the EPOS model was externally validated twice, the validation cohorts were small, which could explain the larger error range in the calibration plots. Therefore, a larger sample multicenter validation study is warranted, considering the latest insights regarding the sample size calculation for the external validation of multivariable prediction models with a binary outcome [50, 51]. Results from this study, such as the outcome event proportion for each ARAT cut-off, could be used in the sample size calculation.

## Conclusions

The EPOS model for predicting upper limb outcome using an ARAT cut-off at 10 points is ready to be tested for its impact in clinical practice in neurologically mild-to-moderately affected first-ever stroke patients with initial upper limb motor impairments who were independent before the stroke, from day 2 onwards. To improve the generalizability of the EPOS model, more external validation studies are needed in large samples with another case-mix and countries other than West European countries. A refined extension of the EPOS model using an ARAT cut-off at 32 points that is serially assessed within the first few weeks poststroke is warranted to differentiate between clinically relevant and better balanced categories of upper limb capacity.

## Supporting information

S1 Checklist STROBE Statement—Checklist of items that should be included in reports of *cohort studies*. (DOCX)

S2 Checklist TRIPOD checklist: Prediction model development and validation. (DOCX)

S1 Fig Receiver operator characteristic curves for the external validation of the EPOS model for upper limb outcome based on the raw data for an ARAT cut-off at 10 points. ARAT, Action Research Arm Test.
(PDF)

S2 Fig Calibration plots for the external validation of the EPOS model for upper limb outcome based on the raw data for an ARAT cut-off at 10 points. The model at day 5 was not externally validated in Cohort 2. The dotted line indicates perfect calibration, meaning that the predicted probabilities by the EPOS model (x-axis) and the observed probabilities in our sample (y-axis) are similar. ARAT, Action Research Arm Test.
(PDF)

S3 Fig Receiver operator characteristic curves for the external validation of the EPOS model for upper limb outcome based on the raw data for an ARAT cut-off at 32 points. ARAT, Action Research Arm Test.
(PDF)

S4 Fig Calibration plots for the external validation of the EPOS model for upper limb outcome based on the raw data for an ARAT cut-off at 32 points. The model at day 5 was not externally validated in Cohort 2. The dotted line indicates perfect calibration, meaning that the predicted probabilities by the EPOS model (x-axis) and the observed probabilities in our sample (y-axis) are similar. ARAT, Action Research Arm Test.
(PDF)

S1 Table Baseline characteristics of included patients of the two validation cohorts. This table includes also patients who were removed from the final analyses, due to missing outcome data, however, patients who died were excluded from the analysis. *, mean (standard deviation); †, median (quartile 1 –quartile 3); ‡, N (%); ARAT, Action Research Arm Test; BI, Barthel Index; FAC, Functional Ambulation Categories; FE, Finger Extension; FMA-UE, Fugl-Meyer Assessment Upper Extremity Subscale; LACS, Lacunar Stroke; MI-LE, Motricity Index Lower Extremity Subscale; MI-UE, Motricity Index Upper Extremity Subscale; mRS, modified Rankin Scale; N, Number; N/A, Not Applicable; N/R, Not Reported; NIHSS, National Institutes of Health Stroke Scale; PACS, Partial Anterior Circulation Stroke; SA, Shoulder Abduction; TACS, Total Anterior Circulation Stroke.
(PDF)

S2 Table Comparison of key baseline characteristics between patients with and without missing data. Mann-Whitney U for ordinal data and Chi-square test for binary data. FAC, Functional Ambulation Categories; FE, Finger Extension; FMA-UE, Fugl-Meyer Assessment Upper Extremity Subscale; LACS, Lacunar Stroke; MI-LE, Motricity Index Lower Extremity Subscale; mRS, modified Rankin Scale; N/A, Not Applicable; NIHSS, National Institutes of Health Stroke Scale; PACS, Partial Anterior Circulation Stroke; SA, Shoulder Abduction; TACS, Total Anterior Circulation Stroke.
(PDF)

S3 Table Discrimination analysis with imputed and raw data for an ARAT cut-off at 10 points. No statistically significant differences in AUC were found between the imputed and raw datasets (p>0.05). Acc, Accuracy; ARAT, Action Research Arm Test; AUC, Area Under the Curve; CI, Confidence Interval; NIR, No Information Rate.
(PDF)

S4 Table Overview of predicted and actual outcome categories with imputed data for an ARAT cut-off at 10 points. ARAT, Action Research Arm Test; N, Number.
(PDF)

S5 Table Overview of predicted and actual outcome categories with imputed data for an ARAT cut-off at 32 points. ARAT, Action Research Arm Test; N, Number.
(PDF)

S6 Table Discrimination analysis with imputed and raw data for an ARAT cut-off at 32 points. No statistically significant differences in AUC were found between the imputed and raw datasets (p>0.05). Acc, Accuracy; ARAT, Action Research Arm Test; AUC, Area Under the Curve; CI, Confidence Interval; NIR, No Information Rate.
(PDF)

S1 Appendix The study’s underlying dataset. (XLSX)
