# Supplementary material for: External validation and extension of the Early Prediction of Functional Outcome after Stroke (EPOS) prediction model for upper limb outcome 3 months after stroke
Source: PLoS One. 2022 Aug 8;17(8):e0272777. doi: 10.1371/journal.pone.0272777 (PMC9359545; doi:10.1371/journal.pone.0272777)
Supplement: S1 Fig — ARAT, Action Research Arm Test. (PDF) [file pone.0272777.s003.pdf]

Fig S1. Receiver operator characteristic curves for the external validation of the EPOS model for upper limb outcome based on the raw data for an ARAT cut-off at 10 points

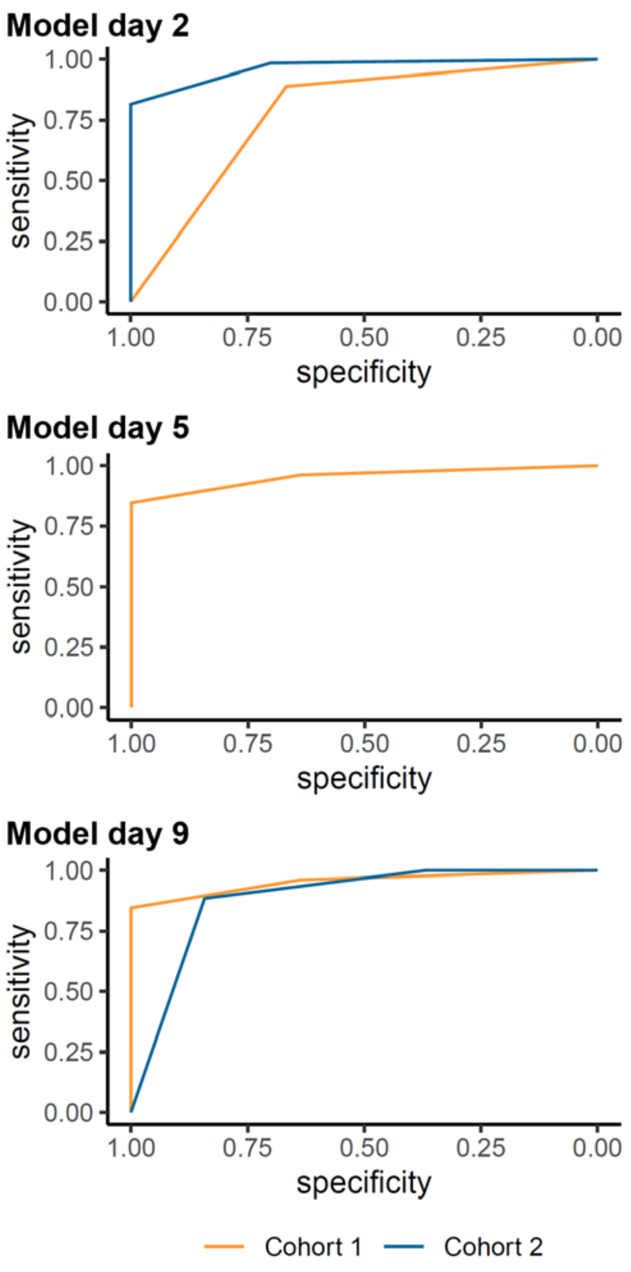

Legend: ARAT, Action Research Arm Test.
