# Supplementary material for: External validation and extension of the Early Prediction of Functional Outcome after Stroke (EPOS) prediction model for upper limb outcome 3 months after stroke
Source: PLoS One. 2022 Aug 8;17(8):e0272777. doi: 10.1371/journal.pone.0272777 (PMC9359545; doi:10.1371/journal.pone.0272777)
Supplement: S1 Table — This table includes also patients who were removed from the final analyses, due to missing outcome data, however, patients who died were excluded from the analysis. *, mean (standard deviation); †, median (quartile 1 –quartile 3); ‡, N (%); ARAT, Action Research Arm Test; BI, Barthel Index; FAC, Functional Ambulation Categories; FE, Finger Extension; FMA-UE, Fugl-Meyer Assessment Upper Extremity Subscale; LACS, Lacunar Stroke; MI-LE, Motricity Index Lower Extremity Subscale; MI-UE, Motricity Index Upper Extremity Subscale; mRS, modified Rankin Scale; N, Number; N/A, Not Applicable; N/R, Not Reported; NIHSS, National Institutes of Health Stroke Scale; PACS, Partial Anterior Circulation Stroke; SA, Shoulder Abduction; TACS, Total Anterior Circulation Stroke. (PDF) [file pone.0272777.s007.pdf]

Table S1. Baseline characteristics of included patients of the two validation cohorts

Characteristic	Validation cohort 1		Validation cohort 2	
	(N=40)	N (%) missing data	(N=90)	N (%) missing data
Age, years†	74.5 (69–78.25)	0 (0)	69 (60.25–77)	0 (0)
Female‡	13 (32.5)	0 (0)	36 (40)	0 (0)
Affected hemisphere, left‡	14 (35)	0 (0)	43 (47.8)	0 (0)
Type of stroke‡		0 (0)		0 (0)
Ischemic	40 (100)		71 (78.9)	
Haemorrhagic	0 (0)		19 (21.1)	
Bamford classification‡		0 (0)		0 (0)
LACS	17 (42.5)		42 (46.7)	
PACS	12 (30)		29 (32.2)	
TACS	11 (27.5)		19 (21.1)	
Thrombolysis, yes‡	16 (40)	0 (0)	17 (18.9)	0 (0)
Thrombectomy, yes‡	16 (40)	0 (0)	27 (30)	0 (0)
Time poststroke†				
Model day 2 (days)	1.04 (0.73–1.37)	0 (0)	3 (2–4)	0 (0)
Model day 5 (days)	7.82 (7.31–8.31)	2 (5)	N/A	N/A
Model day 9 (days)	7.82 (7.31–8.31)	2 (5)	9 (8–10)	5 (5.6)
Clinical scales baseline				
NIHSS (0–42)†	9.5 (5.75–13.25)	0 (0)	7 (4–11)	1 (1.1)
Cognitive disturbance, yes‡				
Inattention	18 (45)	0 (0)	23 (25.6)	0 (0)
Disorientation	15 (37.5)	0 (0)	23 (25.6)	0 (0)
Sensation deficits, yes‡	21 (52.5)	0 (0)	42 (46.7)	0 (0)
Visual impairment, yes‡				
Hemianopia	6 (15)	0 (0)	26 (28.9)	0 (0)
Deviation conjugee	13 (32.5)	0 (0)	17 (18.9)	0 (0)
MI-UE (0–100)†	36 (0–61)	0 (0)	50 (20–65)	0 (0)
MI-LE (0–100)†	37 (20.5–58)	1 (2.5)	42 (29–75)	0 (0)
FMA-UE (0–66)†	10 (4-23)	1 (2.5)	22.5 (7–37)	0 (0)
FAC (0–5)†	0 (0–0)	0 (0)	1 (0–2)	0 (0)
ARAT (0–57)†	N/A	N/A	N/A	0 (0)
mRS (0–5)†	5 (4–5)	0 (0)	4 (4–5)	0 (0)
Predictors				
Model day 2				
FE, yes‡	21 (52.5)	0 (0)	59 (65.6)	0 (0)
SA, yes‡	28 (70)	0 (0)	74 (82.2)	0 (0)
Model day 5				
FE, yes‡	22 (57.9)	2 (5)	N/A	N/A
SA, yes‡	30 (78.9)	2 (5)	N/A	N/A
Model day 9				
FE, yes‡	22 (57.9)	2 (5)	60 (66.7)	6 (6.7)
SA, yes‡	30 (78.9)	2 (5)	76 (84.4)	6 (6.7)

Legend: This table includes also patients who were removed from the final analyses, due to missing outcome data, however, patients who died were excluded from the analysis. *, mean (standard deviation); †, median (quartile 1 – quartile 3); ‡, N (%); FAC, Functional Ambulation Categories; FE, Finger Extension; FMA-UE, Fugl-Meyer Assessment Upper Extremity Subscale; LACS, Lacunar Stroke; MI-LE, Motricity Index Lower Extremity Subscale; MI-UE, Motricity Index Upper Extremity Subscale; mRS, modified Rankin Scale;

N, Number; N/A, Not Applicable; NIHSS, National Institutes of Health Stroke Scale; PACS, Partial Anterior Circulation Stroke; SA, Shoulder Abduction; TACS, Total Anterior Circulation Stroke.
