# Supplementary material for: External validation and extension of the Early Prediction of Functional Outcome after Stroke (EPOS) prediction model for upper limb outcome 3 months after stroke
Source: PLoS One. 2022 Aug 8;17(8):e0272777. doi: 10.1371/journal.pone.0272777 (PMC9359545; doi:10.1371/journal.pone.0272777)
Supplement: S2 Table — Mann-Whitney U for ordinal data and Chi-square test for binary data. FAC, Functional Ambulation Categories; FE, Finger Extension; FMA-UE, Fugl-Meyer Assessment Upper Extremity Subscale; LACS, Lacunar Stroke; MI-LE, Motricity Index Lower Extremity Subscale; mRS, modified Rankin Scale; N/A, Not Applicable; NIHSS, National Institutes of Health Stroke Scale; PACS, Partial Anterior Circulation Stroke; SA, Shoulder Abduction; TACS, Total Anterior Circulation Stroke. (PDF) [file pone.0272777.s008.pdf]

Table S2. Comparison of key baseline characteristics between patients with and without missing data

Characteristic	Validation cohort 1	Validation cohort 2
	**P-value**	**P-value**
Age, years	0.938	0.653
Gender, female/ male	1.000	1.000
Affected hemisphere, left/ right	0.571	0.628
Type of stroke, ischemic/ haemorrhagic	N/A	1.000
Bamford classification, LACS/ PACS/ TACS	0.509	0.157
Thrombolysis, yes/ no	0.713	0.234
Thrombectomy, yes/ no	1.000	0.714
Time poststroke model day 2	0.878	0.380
Clinical scales baseline		
NIHSS (0–42)	0.918	0.397
MI-UE (0–100)	0.775	0.872
MI-LE (0–100)	0.916	0.637
FMA-UE (0–66)	0.544	0.437
FAC (0–5)	0.398	0.134
mRS (0–5)	0.203	0.469
Predictors model day 2		
FE, yes/ no	1.000	0.505
SA, yes/ no	1.000	1.000

Legend: Mann-Whitney U for ordinal data and Chi-square test for binary data. FAC, Functional Ambulation Categories; FE, Finger Extension; FMA-UE, Fugl-Meyer Assessment Upper Extremity Subscale; LACS, Lacunar Stroke; MI-LE, Motricity Index Lower Extremity Subscale; MI-UE, Motricity Index Upper Extremity Subscale; mRS, modified Rankin Scale; N/A, Not Applicable; NIHSS, National Institutes of Health Stroke Scale; PACS, Partial Anterior Circulation Stroke; SA, Shoulder Abduction; TACS, Total Anterior Circulation Stroke.
