# Supplementary material for: External validation and extension of the Early Prediction of Functional Outcome after Stroke (EPOS) prediction model for upper limb outcome 3 months after stroke
Source: PLoS One. 2022 Aug 8;17(8):e0272777. doi: 10.1371/journal.pone.0272777 (PMC9359545; doi:10.1371/journal.pone.0272777)
Supplement: S4 Table — ARAT, Action Research Arm Test; N, Number. (PDF) [file pone.0272777.s010.pdf]

Table S4. Overview of predicted and actual outcome categories with imputed data for an ARAT cut-off at 10 points

	True negatives, N	False negatives, N	True positives, N	False positives, N
Cohort 1				
Model day 2	8	3	24	4
Model day 5	7	1	26	5
Model day 9	7	1	26	5
Cohort 2				
Model day 2	14	1	64	6
Model day 9	8	0	65	12

Legend: ARAT, Action Research Arm Test; N, Number.
