# Supplementary material for: External validation and extension of the Early Prediction of Functional Outcome after Stroke (EPOS) prediction model for upper limb outcome 3 months after stroke
Source: PLoS One. 2022 Aug 8;17(8):e0272777. doi: 10.1371/journal.pone.0272777 (PMC9359545; doi:10.1371/journal.pone.0272777)
Supplement: S5 Table — ARAT, Action Research Arm Test; N, Number. (PDF) [file pone.0272777.s011.pdf]

Table S5. Overview of predicted and actual outcome categories with imputed data for an ARAT cut-off at 32 points

	**True negatives, N**	**False negatives, N**	**True positives, N**	**False positives, N**
Cohort 1				
Model day 2	9	2	22	6
Model day 5	8	0	24	7
Model day 9	8	0	24	7
Cohort 2				
Model day 2	15	0	56	14
Model day 9	8	0	56	21

Legend: ARAT, Action Research Arm Test; N, Number.
