# Supplementary material for: External validation and extension of the Early Prediction of Functional Outcome after Stroke (EPOS) prediction model for upper limb outcome 3 months after stroke
Source: PLoS One. 2022 Aug 8;17(8):e0272777. doi: 10.1371/journal.pone.0272777 (PMC9359545; doi:10.1371/journal.pone.0272777)
Supplement: S6 Table — No statistically significant differences in AUC were found between the imputed and raw datasets (p>0.05). Acc, Accuracy; ARAT, Action Research Arm Test; AUC, Area Under the Curve; CI, Confidence Interval; NIR, No Information Rate. (PDF) [file pone.0272777.s012.pdf]

Table S6. Discrimination analysis with imputed and raw data for an ARAT cut-off at 32 points

	**Validation cohort 1**		**Validation cohort 2**	
	Imputed data	Raw data	Imputed data	Raw data
Model day 2	N=39	N=39	N=85	N=85
Accuracy (95% CI)	0.79 (0.64, 0.91)	0.79 (0.64, 0.91)	0.84 (0.74, 0.91)	0.84 (0.74, 0.91)
Sensitivity	0.92 (0.73, 0.99)	0.92 (0.73, 0.99)	1.00 (0.94, 1.00)	1.00 (0.94, 1.00)
Specificity	0.60 (0.32, 0.84)	0.60 (0.32, 0.84)	0.52 (0.33, 0.71)	0.52 (0.33, 0.71)
Positive predictive value	0.79 (0.59, 0.92)	0.79 (0.59, 0.92)	0.80 (0.69, 0.89)	0.80 (0.69, 0.89)
Negative predictive value	0.82 (0.48, 0.98)	0.82 (0.48, 0.98)	1.00 (0.78, 1.00)	1.00 (0.78, 1.00)
No information rate	0.62 (0.45, 0.77)	0.62 (0.45, 0.77)	0.66 (0.55, 0.76)	0.66 (0.55, 0.76)
P-Value [Acc > NIR]	0.014	0.014	<0.001	<0.001
AUC (95% CI)	0.82 (0.68, 0.95)	0.82 (0.68, 0.95)	0.90 (0.83, 0.97)	0.90 (0.83, 0.97)
Model day 5	N=39	N=37		
Accuracy (95% CI)	0.82 (0.66, 0.92)	0.84 (0.68, 0.94)		
Sensitivity	1.00 (0.86, 1.00)	1.00 (0.85, 1.00)		
Specificity	0.53 (0.27, 0.78)	0.57 (0.29, 0.82)		
Positive predictive value	0.77 (0.59, 0.90)	0.79 (0.61, 0.92)		
Negative predictive value	1.00 (0.63, 1.00)	1.00 (0.63, 1.00)		
No information rate	0.62 (0.45, 0.77)	0.62 (0.45, 0.775)		
P-Value [Acc > NIR]	0.005	0.004		
AUC (95% CI)	0.95 (0.87, 1.00)	0.95 (0.87, 1.00)		
Model day 9	N=39	N=37	N=85	N=80
Accuracy (95% CI)	0.82 (0.66, 0.92)	0.84 (0.68, 0.94)	0.75 (0.65, 0.84)	0.74 (0.63, 0.83)
Sensitivity	1.00 (0.86, 1.00)	1.00 (0.85, 1.00)	1.00 (0.94, 1.00)	1.00 (0.93, 1.00)
Specificity	0.53 (0.27, 0.79)	0.57 (0.29, 0.82)	0.28 (0.13, 0.47)	0.25 (0.11, 0.45)
Positive predictive value	0.77 (0.59, 0.90)	0.79 (0.60, 0.92)	0.73 (0.61, 0.82)	0.71 (0.59, 0.81)
Negative predictive value	1.00 (0.63, 1.00)	1.00 (0.63, 1.00)	1.00 (0.63, 1.00)	1.00 (0.59, 1.00)
No information rate	0.62 (0.45, 0.77)	0.62 (0.45, 0.78)	0.66 (0.55, 0.76)	0.65 (0.54, 0.75)
P-Value [Acc > NIR]	0.005	0.004	0.041	0.061
AUC (95% CI)	0.95 (0.87, 1.00)	0.95 (0.87, 1.00)	0.86 (0.77, 0.95)	0.86 (0.78, 0.95)

Legend: No statistically significant differences in AUC were found between the imputed and raw datasets ($p>0.05$). Acc, Accuracy; ARAT, Action Research Arm Test; AUC, Area Under the Curve; CI, Confidence Interval; NIR, No Information Rate.
